# The impact of Magnetic Resonance Imaging (MRI) on ischemic stroke detection and incidence: minimal impact within a population-based study

**DOI:** 10.1186/s12883-015-0421-2

**Published:** 2015-09-25

**Authors:** Dawn Kleindorfer, Jane Khoury, Kathleen Alwell, Charles J. Moomaw, Daniel Woo, Matthew L. Flaherty, Opeolu Adeoye, Simona Ferioli, Pooja Khatri, Brett M. Kissela

**Affiliations:** Cincinnati Children’s Hospital Medical Center, Department of Pediatrics, 3333 Burnet Avenue, Cincinnati, OH 45229 USA; Department of Neurology, University of Cincinnati, 260 Stetson Street, Suite 2300, Cincinnati, OH 45267-0525 USA; Department of Emergency Medicine, University of Cincinnati, 231 Albert Sabin Way, ML 0769, Cincinnati, OH 45267 USA

**Keywords:** Acute stroke, Epidemiology, Incidence, Brain imaging

## Abstract

**Background:**

There are several situations in which magnetic resonance imaging (MRI) might impact whether an cerebrovascular event is considered a new stroke. These include clinically non-focal events with positive imaging for acute cerebral infarction, and worsening of older symptoms without evidence of new infarction on MRI. We sought to investigate the impact of MRI on stroke detection and stroke incidence, by describing agreement between a strictly clinical definition of stroke and a definition based on physician opinion, including MRI imaging findings.

**Methods:**

All hospitalized strokes that occurred in five Ohio and Northern Kentucky counties (population 1.3 million) in the calendar year of 2005 were identified using ICD-9 discharge codes 430–436. The two definitions used were: “clinical case definition” which included sudden onset focal neurologic symptoms referable to a vascular territory for >24 h, compared to the “best clinical judgment of the physician definition”, which considers all relevant information, including neuroimaging findings. The 95 % confidence intervals (CI) for the incidence rates were calculated assuming a Poisson distribution. Rates were standardized to the 2000 U.S. population, adjusting for age, race, and sex, and included all age groups.

**Results:**

There were 2403 ischemic stroke events in 2269 patients; 1556 (64 %) had MRI performed. Of the events, 2049 (83 %) were cases by both definitions, 185 (7.7 %) met the clinical case definition but were non-cases in the physician’s opinion and 169 (7.0 %) were non-cases by clinical definition but were cases in the physician’s opinion. There was no significant difference in the incidence rates of first-ever or total ischemic strokes generated by the two different definitions, or when only those with MRI imaging were included.

**Conclusions:**

We found that MRI findings do not appear to substantially change stroke incidence estimates, as the strictly clinical definition of stroke did not significantly differ from a definition that included imaging findings. Including MRI in the case definition “rules out” almost the same number of strokes as it “rules in”.

## Background

Magnetic resonance imaging (MRI) has been increasingly used in the diagnostic work-up of ischemic stroke patients since it was introduced in 1988. MRI is more sensitive than standard computed tomography (CT) for detecting a hyper-acute event [1, 2]. In addition, with diffusion-weighted imaging (DWI), MRI can distinguish new events within a previously damaged territory of brain more easily than CT [3–5]. This makes MRI a valuable clinical tool for diagnosing ischemic stroke events. Furthermore, technological improvements, along with reductions in costs, have allowed greater access to MRI across the United States [4]. Within our large, biracial population of 1.3 million, we have reported that use of MRI for diagnosis of ischemic stroke patients had increased significantly between two study periods, from 19 % in 1993/94 to 28 % in 1999 and 57 % in 2005 (*p* < 0.0001) [6, 7].

Surveillance of ischemic stroke incidence is critical for the planning, implementation, and evaluation of new stroke preventative strategies, treatments, and public health activities. However, as new diagnostic technologies are introduced over time, avoiding detection bias in determining temporal trends of stroke incidence can be challenging. For example, consider the case of a patient who presents only with non-focal symptoms, such as confusion. Due to the lack of focal symptoms, this event would not have been counted towards ischemic stroke incidence according to the clinical definition of stroke in past assessments of stroke incidence. However, with an MRI that detected a small positive acute infarct, this event could be “ruled in” as an ischemic stroke by imaging, despite the lack of focality. By contrast, consider the case of a patient who presents with focal symptoms, but with negative imaging. Prior to the advent of MRI, this event would be counted toward stroke incidence because of the focal symptoms. However, a negative MRI might suggest that the event represents worsening of an old infarct or a diagnosis other than stroke, and thus the event would be “ruled out” by imaging. Because of the conflict that results by using a strictly clinical definition of stroke versus using a definition based on imaging, we sought to describe the impact of MRI on the detection of acute ischemic stroke events within a population-based epidemiologic study of stroke incidence.

## Methods

The Greater Cincinnati/Northern Kentucky (GCNK) region includes two southern Ohio counties and three contiguous Northern Kentucky counties that border the Ohio River. Only residents of the five study counties are considered for case ascertainment. There were 17 hospitals in the GCNK region 2005. Previous studies have documented that residents of the five counties who have a stroke exclusively seek care at these hospitals rather than at hospitals in the outlying region [8]. This study was approved by the Institutional Review Boards and included a waiver for individual informed consent: University of Cincinnati, Tri-Health, The Jewish Hospital/Mercy Hospital System, The Christ Hospital, and the St. Elizabeth Healthcare.

The GCNK Stroke Study involved ascertainment of all stroke events that occurred in the population in calendar year 2005. Details of the previous study periods’ case ascertainment have been previously published [6]. In 2005, screening was identical to the techniques used in previous study periods. Study nurses abstracted the medical records of all area residents who were either inpatients or discharged from the emergency department with primary or secondary stroke-related International Classification of Disease, 9th Revision (ICD-9) discharge diagnoses 430–436 at the 17 acute-care hospitals in the study region. Stroke cases not found by this process were ascertained via screening of all stroke-related visits to the region’s 9 public health clinics and 7 hospital-based outpatient clinics and family practice centers. Strokes listed as the primary or secondary cause of death by one of the five county coroners’ offices were also included. Further monitoring involved examination of records of potential stroke cases in a random sample of 51 of the 832 primary care physicians’ offices and 25 of the 126 nursing homes in the GCNK region. Sampling was necessary given the large number of physician offices and nursing homes in the region. Sites were selected randomly by the study statistician from a list generated from a combination of the local yellow pages and the American Medical Association listing of physicians in the region. All events were cross-checked to prevent double counting.

Once potential cases were identified, a study research nurse abstracted information regarding stroke symptoms, physical exam findings, past medical/surgical history, medication use prior to stroke, social history/habits, pre-hospital evaluation, vital signs and emergency room evaluation, neurological evaluation, diagnostic test results (including lab testing, EKG and cardiac testing, and neuroimaging of any type), treatments, and outcome. Stroke severity was estimated via a validated method of retrospective NIH Stroke Scale score (rNIHSS) obtained from review of the physician exam as documented in the emergency department evaluation [9]. Classification of race/ethnicity was as self-reported in the medical administrative record. The research nurse made a determination as to whether a stroke or TIA may have occurred. Nurse abstractors were instructed to consult with study physicians for any questionable cases. If the nurse abstractor was unsure whether or not a stroke occurred, the event was abstracted so a study physician could determine whether or not the event was a stroke. Both study nurses and study physicians undergo extensive training prior to reviewing events, and the study maintains detailed physician and research nurse study manuals that describe screening, abstraction, and reviewing procedures, ensuring a continuity of methodology between study personnel.

Cases of acute ischemic strokes, both first-ever and recurrent, were included in the present analysis. Intracerebral hemorrhage (ICH) and subarachnoid hemorrhage (SAH) events were not included. The onset of stroke symptoms must have occurred within the study time period. Charts were screened for an additional 60 days beyond the end of the study periods to capture patients who suffered a stroke during the study period but had not yet been discharged.

A stroke-trained study physician reviewed every abstract to verify whether a stroke or TIA had occurred. Two definitions were used to identify ischemic stroke cases in 2005. The first definition was a purely clinical definition: a must have had focal neurologic deficit in a defined vascular territory lasting >24 h (definition adapted from the Classification for Cerebrovascular Diseases III [10] and from epidemiological studies of stroke in Rochester, MN [11]). The clinically-defined TIA, in which symptom duration is less than 24 h, is therefore excluded from this definition. Imaging results were not considered for this clinical definition. For the second definition, physicians were asked to make a separate judgment about whether or not an ischemic stroke had occurred, after taking into account all available information, including imaging reports and, when necessary, review of actual images. For this definition, events with transient symptoms with positive DWI imaging are considered ischemic strokes [12]. The comparison of these two definitions is the subject of this analysis.

Data were managed and analyzed using SAS versions 8.02 and 9.2, respectively (SAS Institute, Cary, NC). All analysis included the sampling weights to account for the out-of-hospital ascertainment sampling plan. Results are presented as raw frequencies with the associated weighted percentages, weighted means and the associated standard error, or weighted medians, as appropriate. Generalized estimating equations (GEE) [13] were used to estimate the means and standard errors correctly; this analytical method also accounted for those patients with more than one event in the time period studied. The denominator for the calculation of incidence rates was extracted from the U.S. Census Bureau website (www.census.gov) for the counties included in our stroke population. The 95 % confidence intervals (CI) for the incidence rates were calculated assuming a Poisson distribution. Rates were standardized to the 2000 U.S. population, adjusting for age, race, and sex, and included all age groups.

## Results and discussion

During 2005, 2403 ischemic stroke events in 2269 patients presented to medical attention, of which 1853 were first-ever ischemic strokes. These events were classified as cases by one or both of the case definitions described in Methods. The demographics of the patients and the strokes are presented in Table 1. Of the 2403 events, we report the following imaging results as the raw number of cases, and (weighted percentage), to account for out-of-hospital ascertainment sampling plan: 1556 (64 %) had an MRI performed with diffusion-weighted imaging, 9 cases had MRI without DWI, and in 6 cases it is unknown whether or not MRIs included DWI, 833 (34 %) had only CT imaging, and 14 cases (1.6 %) had no brain imaging at all. In all three study periods, > 95 % of ischemic cases had a head CT performed.

**Table 1:** Demographics of first and recurrent ischemic stroke patients, and for strokes GCNK region, 2005

	Ischemic stroke patients *n* = 2,269^a,^ ^b^
Age in years, mean ± SEM	70.2 ± 0.35
Black, *N* (weighted %)	492 (20.1 %)
% Female, *N* (weighted %)	1,266 (55.4 %)
Pre-stroke disability (mRS ≥ 2), *N* (weighted %)	1142 (47.7 %)
History of prior stroke, *N* (weighted %)	429 (19.5 %)
retrospective NIHSSS, median (25th, 75th percentile)	3 (1, 7)

^a^ = Data are presented as weighted mean and standard error, raw n (weighted percentage) and weighted median (25th, 75th percentile)

^b^ = For each patient, these summary statistics pertain to the first ischemic stroke in study period; 2,148 patients had only 1 event, 108 patients had 2 events, and 13 patients had 3 events

The two case-definitions (clinical vs. physician judgment) were in agreement for 2049 events (83 % of total events). However, 185 events (7.7 %) were non-cases by clinical definition but were considered events by physician judgment. These events occurred mostly in those who presented with non-focal symptoms (*n* = 128), but they also occurred in cases initially classified as TIAs by clinical definition (*n* = 57). There were also 169 events (7.0 %) were cases by clinical definition but were non-cases by physician judgment. These events uniformly had a reported negative DWI, and often reflected other diagnoses with focal symptoms, such as migraine or seizure. Therefore, inclusion of MRI had a net effect of ruling out almost exactly the same amount of strokes as it ruled in, using the physician judgment definition. Table 2 shows the proportions of MRI utilization and DWI positivity for the two case definitions.

**Table 2:** Rates of utilization of MRI for ischemic stroke events, and rates of DWI positivity, by case definition

Case definition	Number of events	MRI with DWI obtained (weighted % of total) ^a^	Positive DWI (weighted % of MRIs)^a^
Clinical definition YES Physician Judgment YES	2,049	1,233 (60 %)	1,102 (82 %)
Clinical definition NO Physician Judgment YES	185	166 (90 %)	165 (99 %)
Clinical definition YES Physician Judgment NO	169	157 (84 %)	0 (0 %)

^a^ = Note the percentages presented are weighted to reflect out-of-hospital sampling

In addition to the 2049 events discussed in the preceding paragraph, there were 11 patients that were classified as non-cases by both definitions despite having positive DWI on imaging. These patients included 7 incidental findings, 3 with diffuse anoxic brain injury, and one that was related to a traumatic injury.

Table 3 examines the association between pre-selected factors which may possibly affect acute clinical decision-making and later case assignment. Only gender, MRI use and associated DWI classification have differing rates between the agreement and disagreement categories. Incidence rates for first-ever and total (i.e., first-ever plus recurrent) ischemic stroke events, for black and white only, were generated by standardizing to age, race, and sex from the 2000 U.S. census. Overall, as shown in Table 4, there was no significant difference in the incidence rates of first-ever or total ischemic strokes generated by the two different definitions.

**Table 3:** Rates of selected factors potentially related to agreement and disagreement of clinical definition and physician judgment of stroke

	Agreement *N* = 2049	Disagreement *N* = 354	*p*-value
Age (<65 years)	677 (31.7 %)	122 (32.8 %)	0.82
Race (Black)	454 (21.3 %)	81 20.7 %)	0.89
Sex (Female)	1121 (53.4 %)	224 (65.5 %)	0.02
Prior Ischemic stroke	470 25.3 %)	80 27.1 %)	0.75
Seen by neurologist	1434 (65.3 %)	243 (66.3 %)	0.94
MRI done	1233 (59.6 %)	323 (86.8 %)	<0.0001
MRI with positive DWI	1102 (82.5 %)	165 (41.9 %)	<0.0001

Data presented as raw n (weighted % of the factor)

**Table 4:** Ischemic stroke incidence rates, black and white only (per 100,000), GCNK region, using clinical only vs. physician judgment definitions of stroke, standardized to US. Census 2000 population, weighted for sampling

	Clinical definition	Physician judgment
All strokes		
First-Ever Ischemic Stroke Incidence*	140 (134, 147)	139 (133, 145)
	*N* = 1687	*N* = 1727
First and Recurrent Stroke event rate*	183 (176, 190)	177 (170, 184)
	*N* = 2206	*N* = 2223
MRI done only		
First-Ever Ischemic Stroke Incidence**	90 (85, 96)	90 (85, 95)
	*N* = 1203	*N* = 1196
First and Recurrent Stroke event rate**	113 (107, 118)	109 (103, 114)
	*N* = 1583	*N* = 1519

Note: restricted to African American and White (12 other race events not included in rates to ensure accurate standardization)

* = *p* = 0.82 for first-ever stroke, *p* = 0.22 for all

** = *p* = 1.00 for first-ever stroke, *p* = 0.32 for all

## Conclusions

We found that use of MRI did not significantly impact ischemic stroke detection within our population. Our hypothesis had been that MRI would detect milder events and therefore would artifactually increase stroke incidence. However, by comparing a strictly clinical definition (requiring focal deficits referable to a vascular distribution lasting > 24 h) and a physician-judgment definition (which used all available information including imaging results), we found that the amount of strokes “ruled out” was roughly equivalent to the number of strokes “ruled in” by MRI. This has important implications for stroke surveillance studies that monitor trends in stroke incidence and mortality over time within an environment with increasing use of neuroimaging.

It should be noted that the physician judgment of a case did not necessarily duplicate the rate of diffusion-weighted imaging changes on MRI. Eleven patients with + DWI were not judged to be cases by either the clinical definition or physician judgment. In addition, 131 cases with negative DWI on MRI were nevertheless called cases by both definitions. Typically, such cases are related to substantial delays in presentation, but some represent “DWI-negative strokes” in the physician’s judgment. Previous studies have found a prevalence of approximately 5 % DWI negative strokes in case series or single-center studies, but up to 25 % if the MRI is done within 24 h [14, 15]. These findings emphasize the importance of clinical interpretation of events in stroke surveillance studies, beyond simply using ICD-9 coding and imaging results.

Transient ischemic attacks (TIAs) present an especially challenging issue when comparing these two definitions. Clinically, a TIA has previously been defined as focal neurologic symptoms lasting <24 h. However, in the “physician’s judgment” definition, transient events with positive DWI are considered ischemic infarcts. This means that 57 cases in our analysis were considered strokes by one definition (judgment) but TIA by the other (clinical). Thus, the ambiguity regarding classification of transient events with positive imaging will have an effect on the surveillance of TIA incidence rates over time. This is especially true now with the new definition of TIA that requires an absence of DWI changes on MRI, [12] which is complicated by the fact that not every evaluation of TIA events includes imaging. A recent analysis by our group found that requiring MRI for every TIA would result in performing more than twice as many MRIs, which would represent a significant additional public health expenditure, most likely without significant changes in clinical management [16].

Lakshminarayan et al. have previously evaluated the impact of varying definitions on stroke incidence in the Minnesota Stroke Survey [17]. In this study, stroke incidence was evaluated every 5 years between 1980 and 2000. During this time period, the utilization of CT changed significantly, from 75 % in 1980 to 98 % in 2000. In their analysis, stroke incidence rates varied widely by definition in the earlier study periods, especially when comparing the strictly clinical definition to the “neuroimaging” definition (which was largely CT-driven); the clinically-defined rates were nearly twice that of the neuroimaging-defined rates. This discrepancy between the definitions was much less in the most recent study period. While it is impossible to know for sure why the study by Lakshminarayan and the current analysis found such a differential impact of imaging, the difference probably lies in the time frames of the two studies. During the 1980s and 1990s, MRI use was infrequent, but CT use was significantly increasing. The authors from the Minnesota Heart Stroke Incidence Study suspected that the use of CT helped to make the stroke-related ICD-9 codes more specific over time. In 2005 for our analysis, stroke care had advanced significantly, [18] and MRI use was much more prevalent. It may be that the diagnosis of stroke was improved by the addition of CT drastically in earlier years, but now MRI does not add that much more incrementally to the accuracy of ICD-9 coding. However, it is also possible that MRI’s ability to “rule out” stroke in the opinion of the investigator counterbalances this effect, something that CT does not do as effectively since it cannot gauge the acuity of the infarct as well. Of course, obtaining the most accurate diagnosis of stroke patients is extremely valuable, as it allows the appropriate treatment of the patient as well as enabling rigorous interpretation of quality data, clinical trials, and policies. Clinicians will need to make their own determinations about the utility of MRI in individual stroke patients, as our findings are not intended to drive clinical practice, and we cannot comment on the cost-effectiveness of MRI as a diagnostic tool.

A significant limitation in our analysis is that not all the patients received an MRI scan for their event. This reflects the true practice of stroke care within a community, as many different types of care settings are represented within our population, including community vs. academic hospitals, and out-of-hospital care. This means that it is possible that some of the events that did not receive an MRI potentially would have been considered a stroke if they had. Any study that requires MRI of all its participants will clearly have a referral bias, and such a requirement would not be possible within a large population. Interestingly, the utilization of MRI does vary by whether the clinical and physician judgment definitions agree: for events where both definitions agreed that they were cases, the use of MRI was only 60 %, but for events where the two definitions disagreed, the rate was much higher. This suggests that MRI is likely used in the more challenging cases, where the diagnosis of stroke is not as obvious. When we analyzed the impact on stroke incidence only among those cases who obtained an MRI, however, we still did not see a significant change in stroke incidence rates.

The potential for bias of incomplete case ascertainment is important to consider in any study that examines incidence of a disease within a population. Our additional use of passive surveillance of emergency rooms, nursing homes, physician offices, and clinics should reduce chances of incomplete ascertainment. In addition, the random sampling of offices and nursing homes assumes a uniform distribution of strokes by region; this of course, may not be the case. However, we believe that our consistent methods and clinical case definition has minimized possible ascertainment biases. In addition, any incidence study that relies on medical contact for counting of events risks missing events that were not recognized by the general public as needing medical attention.

It appears that the increasing utilization of MRI will likely have little impact on the overall incidence and event rates for stroke. However, for other more clinical analyses, the physician’s judgment definition will be more relevant than the pure clinical definition, as it would seem to better account for stroke mimics, non-events, and non-focal infarcts.

## Abbreviations

MRI: Magnetic resonance imaging
ICD-9: International Classification of Diseases, version 9
CI: Confidence intervals
CT: Computed tomography
DWI: Diffusion weighted imaging
GCNK: Greater Cincinnati Northern Kentucky
EKG: Electrocardiogram
rNIHSS: Retrospective National Institutes of Health Stroke Scale
TIA: Transient ischemic attack
ICH: Intracerebral hemorrhage
SAH: Subarachnoid hemorrhage
GEE: Generalized estimating equations

## References

[1] Camilo O, Goldstein LB (2003). Statewide assessment of hospital-based stroke prevention and treatment services in north carolina: changes over the last 5 years. Stroke.

[2] Caveney AF, Silbergleit R, Frederiksen S, Meurer WJ, Hickenbottom SL, Smith RW (2010). Resource utilization and outcome at a university versus a community teaching hospital in tpa treated stroke patients: a retrospective cohort study. BMC Health Serv Res.

[3] Jewel KL (1985). Sensitivity of mri vs. Ct for neuroimaging. AJR Am J Roentgenol.

[4] Kidwell CS, Wintermark M (2010). The role of ct and mri in the emergency evaluation of persons with suspected stroke. Curr Neurol Neurosci Rep.

[5] Chalela JA, Kidwell CS, Nentwich LM, Luby M, Butman JA, Demchuk AM (2007). Magnetic resonance imaging and computed tomography in emergency assessment of patients with suspected acute stroke: a prospective comparison. Lancet.

[6] Kleindorfer D, Broderick J, Khoury J, Flaherty M, Woo D, Alwell K (2006). The unchanging incidence and case-fatality of stroke in the 1990s: a population-based study. Stroke.

[7] Kissela BM, Khoury JC, Alwell K, Moomaw CJ, Woo D, Adeoye O (2012). Age at stroke: temporal trends in stroke incidence in a large, biracial population. Neurology.

[8] Broderick J, Brott T, Kothari R, Miller R, Khoury J, Pancioli A (1998). The greater Cincinnati/Northern Kentucky stroke study: preliminary first-ever and total incidence rates of stroke among blacks. Stroke.

[9] Williams LS, Yilmaz EY, Lopez-Yunez AM (2000). Retrospective assessment of initial stroke severity with the NIH Stroke Scale. Stroke.

[10] (NINDS) NIoNDaS (1990). Classification of neurological disorders iii. Stroke.

[11] Brown R, Whisnant J, Sicks J, O'Fallon W, Wiebers D (1996). Stroke incidence, prevalence, and survival: secular trends in Rochester, Minnesota, through 1989. Stroke.

[12] Easton JD, Saver JL, Albers GW, Alberts MJ, Chaturvedi S, Feldmann E (2009). Definition and evaluation of transient ischemic attack: a scientific statement for healthcare professionals from the American Heart Association/American Stroke Association stroke council; council on cardiovascular surgery and anesthesia; council on cardiovascular radiology and intervention; council on cardiovascular nursing; and the interdisciplinary council on peripheral vascular disease. The American academy of neurology affirms the value of this statement as an educational tool for neurologists. Stroke.

[13] Zeger SL, Liang KY (1986). Longitudinal data analysis for discrete and continuous outcomes. Biometrics.

[14] Sylaja PN, Coutts SB, Krol A, Hill MD, Demchuk AM (2008). When to expect negative diffusion-weighted images in stroke and transient ischemic attack. Stroke.

[15] Oppenheim C, Stanescu R, Dormont D, Crozier S, Marro B, Samson Y (2000). False-negative diffusion-weighted mr findings in acute ischemic stroke. AJNR Am J Neuroradiol.

[16] Adeoye OHL, Moomaw CJ, Alwell KA, Khoury J, Woo D, Flaherty ML (2010). How much would performing diffusion-weighted imaging for all transient ischemic attacks increase mri imaging utilization?. Stroke.

[17] Lakshminarayan K, Anderson DC, Jacobs DR, Barber CA, Luepker RV (2009). Stroke rates: 1980-2000: The Minnesota stroke survey. Am J Epidemiol.

[18] Schwamm L, Fayad P, Acker JE, Duncan P, Fonarow GC, Girgus M (2010). Translating evidence into practice: a decade of efforts by the American Heart Association/American Stroke Association to reduce death and disability due to stroke: a presidential advisory from the American Heart Association/American Stroke Association. Stroke.

